# Optic cup morphogenesis across species and related inborn human eye defects

**DOI:** 10.1242/dev.200399

**Published:** 2023-01-30

**Authors:** Marcos J. Cardozo, Elena Sánchez-Bustamante, Paola Bovolenta

**Affiliations:** ^1^ Centro de Biología Molecular Severo Ochoa, Consejo Superior de Investigaciones Científicas-Universidad Autónoma de Madrid, c/ Nicolás Cabrera 1, Cantoblanco, Madrid 28049, Spain; ^2^ Centro de Investigación Biomédica en Red de Enfermedades Raras (CIBERER), c/ Nicolás Cabrera 1, Cantoblanco, Madrid 28049, Spain

**Keywords:** Neural retina, Retina pigment epithelium, Organoids, Zebrafish, Human, Mammals, Anophthalmia, Microphthalmia, Gene regulatory networks, Cavefish

## Abstract

The vertebrate eye is shaped as a cup, a conformation that optimizes vision and is acquired early in development through a process known as optic cup morphogenesis. Imaging living, transparent teleost embryos and mammalian stem cell-derived organoids has provided insights into the rearrangements that eye progenitors undergo to adopt such a shape. Molecular and pharmacological interference with these rearrangements has further identified the underlying molecular machineries and the physical forces involved in this morphogenetic process. In this Review, we summarize the resulting scenarios and proposed models that include common and species-specific events. We further discuss how these studies and those in environmentally adapted blind species may shed light on human inborn eye malformations that result from failures in optic cup morphogenesis, including microphthalmia, anophthalmia and coloboma.

## Teaser

**Summary:** This Review analyses the common principles by which vertebrates generate overall similar cup-shaped eyes, pointing to relevant differences and discussing the relationship with congenital eye malformations.

## Introduction

Sight is a fundamental physical sense that allows almost all animal species to reconstruct the surrounding world and interact with it. However, different species require different visual abilities adapted for their lifestyle, behaviour and ecological niche. For example, being diurnal or nocturnal, terrestrial or marine, or being a predator or prey, imposes very different visual needs. These needs have been behind the evolutionary pressures that culminate in the emergence of different visual system configurations (Baden, 2020; Vallerga, 1994), with the eye as one of the most striking examples of variability through a multibranched evolution (Gehring, 2014; Goldsmith, 1990; Lamb et al., 2007; Martinez-Morales and Locascio, 2016).

The eye is the primary visual organ and receives light information through two types of cells: photoreceptors and pigmented cells. This basic unit is thought to constitute the ancestral prototypic eye, from which all existing eyes have arisen (i.e. eye spots, ocellus, compound eyes, eye cups or camera eyes; see Table 1; Arendt, 2003; Gehring, 2014; Goldsmith, 1990; Martinez-Morales and Locascio, 2016). The ‘camera’ eye is perhaps the most sophisticated among the existing light-capturing structures. Its evolutionary origin implies the acquisition of a refractive and transparent lens though which the light is focused and projected into the retina: the neural structure that processes and transmits light information to the brain (Baden, 2020; Vallerga, 1994). Camera eyes are found in all vertebrates and in some invertebrates, such as spiders, cnidarians and cephalopods (Lamb et al., 2007). Despite different adaptations, all camera eyes have a hemispheric or ‘cup’ shape, which provides a better visual resolution than any other existing eye designs (Lamb et al., 2007; Martinez-Morales and Locascio, 2016).

**Table 1.**
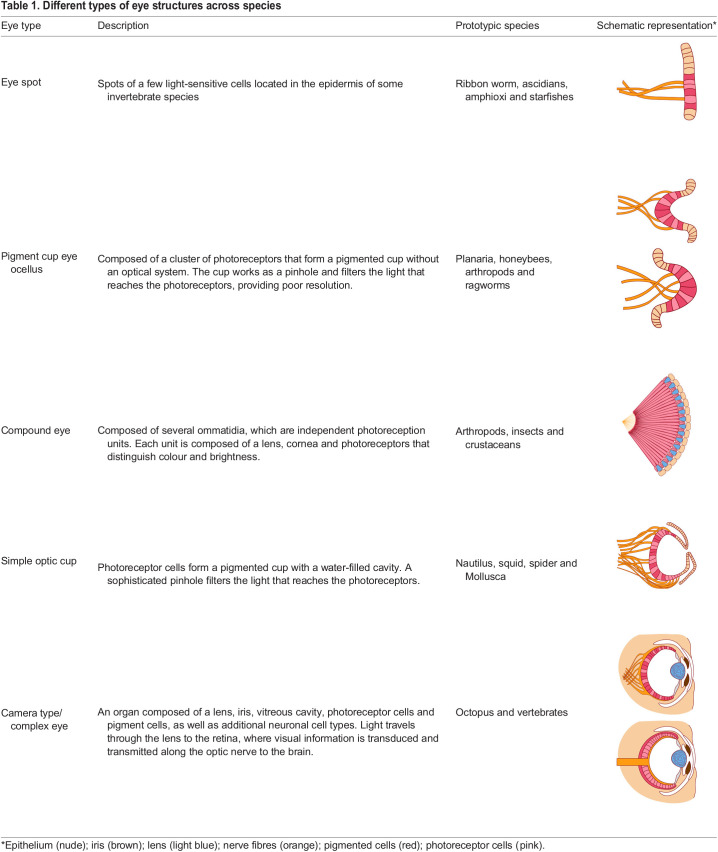
Different types of eye structures across species

**Fig. 1.**
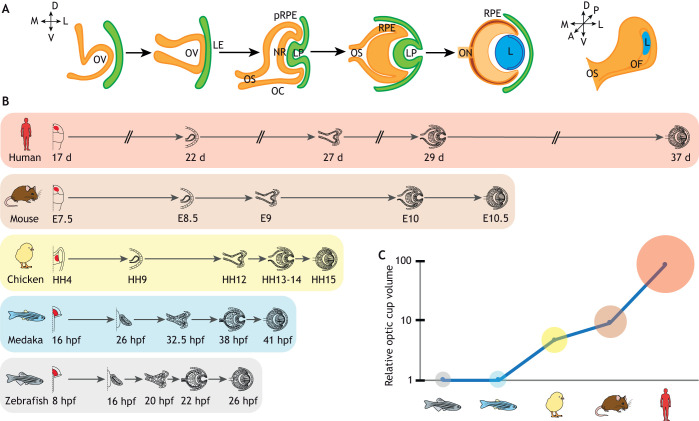
**Schematic representation of vertebrate eye morphogenesis according to a species-specific schedule.** (A) Steps of eye morphogenesis from the initial optic vesicle (OV) to a fully formed optic cup (OC). Lens tissue is depicted in green; neural derivatives in orange. The fully formed lens (L) is represented in blue. LE, lens ectoderm; LP, lens placode; NR, neural retina; OC, optic cup; OF, optic fissure; ON, optic nerve; OS, optic stalk; pRPE, presumptive retinal pigment epithelium; RPE, retinal pigment epithelium. (B) Timelines of eye development in different vertebrate species. (C) Graph representing the optic cup volume relative to that of zebrafish in the different depicted species: (from the right) human, mouse, chicken, medaka and zebrafish.

Past studies have established that, in all vertebrates, the acquisition of this cup shape starts with specification of the eye field (see Glossary, Box 1) in the mid anterior neural plate, followed by the bilateral protrusion of this region into two optic vesicles (OVs), which thereafter fold, forming the optic cups (OCs) (Fig. 1A) (Martinez-Morales et al., 2017). This folding leads to the generation of a transient groove along the ventral pole of the eye rudiment called the optic or choroid fissure (OF; see Glossary, Box 1), which extends along the optic stalk (Fig. 1A; see Glossary, Box 1), the structure that connects the OC to the adjacent neural tube, thereafter forming the optic nerve (see Glossary, Box 1). The OF enables the ingression of cells from periocular mesenchyme (POM) (see Glossary, Box 1) that generate the retinal vasculature and the egression of retinal axons. Thereafter, the borders of the OF fuse forming the optic nerve (Gestri et al., 2018; Morcillo et al., 2006).

**Box 1. Glossary**

**Eye or retinal field.** The region of the anterior neural plate that comprises the precursors of the neural components of the eye.

**Lens ectoderm.** Ectoderm derived from the pre-placodal region and abutting the optic vesicle.

**Lens placode.** A thickening of the lens ectoderm that serves as the precursor to the lens.

**Neural retina.** The light-sensitive tissue of eye, composed of different types of neurons, including photoreceptors and retinal ganglion cells, organized in interconnected layers and responsible of transmitting light information to the brain.

**Optic disc.** Also known as the blind spot of the retina, this is the region where the optic fibres converge to become part of the optic nerve and represents the interface between the optic stalk and the neural retina.

**Optic fissure.** The groove along the ventral region of the optic vesicle that enfolds the axons of the retinal ganglion cells, leaving the eye and the mesenchymal cells that ingress to form the hyaloid artery. This structure is also named the choroid fissure.

**Optic nerve.** The structure derived from the optic stalk and mostly composed of the axons of the retinal ganglion cell layer carrying visual information to the brain.

**Optic stalk.** A derivative of the ventral optic vesicle that connects the vesicle with the neural tube.

**Periocular mesenchyme.** Mesenchymal cells surrounding the developing eye and contributing, among others, to the development of the anterior ocular segments.

**Retinal pigmented epithelium.** The epithelial layer composed of pigmented cells that surrounds the neural retina and supports photoreceptor function.

The correct execution of all these morphogenetic events depends on the reiterative use of a conserved core set of regulatory molecules, including transcription factors and morphogenetic signalling pathways that collectively form specific gene regulatory networks (GRNs). These GRNs are evolutionarily conserved, although the specific interaction between some of their components may vary to fulfil species-specific visual perception needs (Beccari et al., 2013; Casares and Almudi, 2016; Chen and Desplan, 2020; Hoshino et al., 2017; Martinez-Morales, 2016). In all, these observations suggest that vertebrate eye morphogenesis should occur following very similar principles and mechanics across phylogeny. Many developmental neurobiologists interested in eye development have thus turned to transparent embryos, such as those of the zebrafish or medaka, to study how the vertebrate eye acquires its shape *in vivo* (Heermann et al., 2015; Kwan et al., 2012; Moreno-Mármol et al., 2021; Nicolas-Perez et al., 2016; Sidhaye and Norden, 2017). The successful development of OC organoids derived from fish, mouse or human embryonic stem cells (ESCs) or induced pluripotent stem cells (iPSCs) have further boosted the studies on the physical forces behind eye morphogenesis, providing additional insights and indicating species-specific features (e.g. Eiraku et al., 2011; Nakano et al., 2012; O'Hara-Wright and Gonzalez-Cordero, 2020; Zilova et al., 2021).

In this Review, we summarize the data obtained from the above studies and analyse the common principles by which vertebrates generate overall similar cup-shaped eyes, but also indicate relevant differences. Indeed, even among very similar vertebrate eyes, there is variability in organ size, time of development (Fig. 1B) and/or regenerative capacities. But how has eye morphogenesis adapted to fulfil this variability? What do animal models tell us about human eye development? Can studies on eye morphogenesis help us to understand how human congenital eye malformations arise? Here, we try to address these questions, focusing on one crucial event in eye formation: the folding of the OV into an OC. We refer the reader to previous comprehensive reviews for information about the main events that precede (Cavodeassi, 2018; Giger and Houart, 2018; Sinn and Wittbrodt, 2013) or follow (Chow and Lang, 2001; Miesfeld and Brown, 2019) this crucial eye developmental step, or about the GRNs that govern them (Beccari et al., 2013; Casares and Almudi, 2016; Chen and Desplan, 2020; Martinez-Morales, 2016).

## Folding of the optic vesicle into an optic cup in fast-developing species

**Fig. 2.**
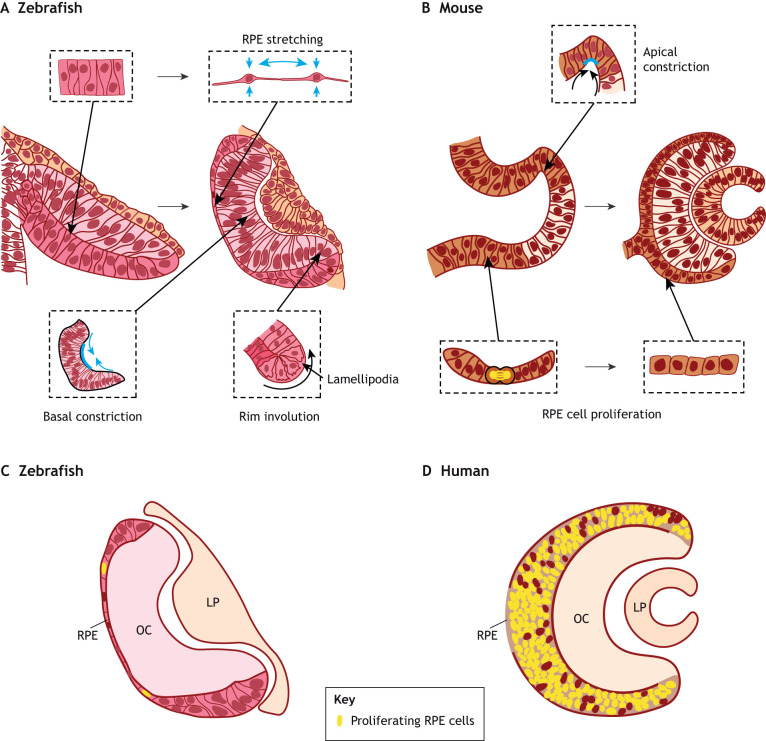
**Comparison between teleost and mammalian optic cup morphogenesis.** (A,B) Schematic representations of the zebrafish (A) and mouse (B) transition from optic vesicle to optic cup (OC). The dashed outlines indicate the shape changes that each cell type undergoes as it acquires the identity of neural retina (NR), retinal pigmented epithelium (RPE) and hinge cells. (C,D) Schematic representations of a section through the zebrafish (C) and human (D) OC, illustrating the differences observed in RPE cell proliferation (green nuclei). LP, lens placode.

In all vertebrate species, the folding of the OV into a cup entails two crucial and concomitant events. First, neuroepithelial precursors acquire two different fates, neural retina (NR; see Glossary, Box 1) and retinal pigment epithelium (RPE) (see Glossary, Box 1), driven by signalling molecules, such as Sonic hedgehog (Shh), fibroblast growth factors (Fgfs) or Wingless-related (Wnt) proteins (Cardozo et al., 2020), which trigger differential transcriptional states in the GRNs controlling cell identity acquisition (Beccari et al., 2013; Buono et al., 2021; Fuhrmann, 2010; Yamada et al., 2021). Second, individual neuroepithelial cells change their shape, giving rise to elongated NR cells, squamous or cuboidal cells of the RPE, and the wedge-shaped cells at the NR-RPE connecting hinges (Figs 1A and 2A).

These events are likely common to all vertebrates, but the morphological appearance of the vesicles and the speed of their morphogenesis are parameters that distinguish many teleosts from amniotes. For example, in zebrafish and medaka, the OV is an elongated and flat bilayer composed of a pseudostratified neuroepithelium (Fig. 2A) that folds into a cup in just a few hours (Fig. 1B). The existence of several mutant lines, the easy embryological manipulations and the transparent nature of their embryos make the zebrafish and medaka ideal models to follow how the eye forms in real time and define the underlying mechanics (Casey et al., 2021).

Cell-autonomous basal constriction of NR progenitors is one of the pillars of vertebrate OV folding (Fig. 2A) based on the discovery of the *ojoplano* (*opo*; ‘flat eye’ in Spanish) medaka mutant, in which the OV remains unfolded (Martinez-Morales et al., 2009). The *opo* gene encodes a transmembrane protein that localizes at the basal end-feet of the NR precursors, controlling the endocytosis of focal adhesion components and their interaction with the actomyosin cytoskeleton (Bogdanović et al., 2012; Martinez-Morales et al., 2009). Pulsatile contraction of the actomyosin cytoskeleton shrinks the basal end-feet of NR cells (Nicolas-Perez et al., 2016; Sidhaye and Norden, 2017), generating a tension that favours OV inward bending, as also confirmed by local interference with myosin II activity (Moreno-Mármol et al., 2021). These events are strongly dependent on basal end-feet laminin and its interaction with the abutting extracellular matrix (ECM) that seems to strengthen contractile forces (Nicolas-Perez et al., 2016). Indeed, genetic inactivation or knock-down of laminin components (*lamc1* and *lama1*) impairs basal contractility and the transmission of mechanical tension, ultimately preventing OV folding (Bryan et al., 2016; Nicolas-Perez et al., 2016).

As the OV bends inwards, the lateral-NR layer keeps growing through the continuous incorporation of neuroepithelial cells from the medial OV layer through a collective migration process (Sidhaye and Norden, 2017; Soans et al., 2022) known as ‘rim involution’ or ‘epithelial flow’ (Heermann et al., 2015; Kwan et al., 2012; Picker et al., 2009; Sidhaye and Norden, 2017). This event is part of broader cellular rearrangements characterized by ‘pinwheel’-like movements that begin with OV evagination, displacing NR and RPE precursors into more posterior positions, and promoting the incorporation of new cells from the neural tube (Kwan et al., 2012). Rim involution takes place mostly at the ventral hinge (Fig. 2A) through the dynamic extrusion of cryptic basal lamellipodia that extend in the direction of migration, attaching to the ECM (Heermann et al., 2015; Kwan et al., 2012; Sidhaye and Norden, 2017; Soans et al., 2022), thereby enabling cellular translocation (Heermann et al., 2015; Sidhaye and Norden, 2017). Notably, the ECM surrounding the OV, in part deposited by periocular neural crest cells (Bryan et al., 2020), undergoes topological changes that are associated with different cellular dynamics, thereby influencing the efficiency of directed collective rim cell migration (Soans et al., 2022). Besides the important role of the surrounding ECM, bone morphogenetic protein (BMP) signalling seems to facilitate this involution (Heermann et al., 2015), which, if disrupted, either by manipulating BMP signalling or by interfering with lamellipodia formation, prevents the acquisition of a proper cup shape. In the absence of cellular translocation, progenitor cells accumulate at the ventral inner layer but they nevertheless acquire NR fate (Heermann et al., 2015; Sidhaye and Norden, 2017), suggesting a fate pre-commitment. Notably, the last cells that translocate into the ventral inner layer and the few that translocate dorsally retain stem cell properties and eventually generate the peripheral rim of the retina or ciliary margins (CMs) (Heermann et al., 2015), which allows the continuous growth of the eye in both amphibians and fish.

Rim involution depletes cells from the inner OV layer, in principle unbalancing the size of the layers. This disequilibrium is minimized by the concomitant spreading of the small patch of remaining outer layer cells, positioned in the dorso-medial OV (Kwan et al., 2012; Li et al., 2000; Moreno-Mármol et al., 2021). This patch of neuroepithelial progenitors is committed to a RPE fate. RPE progenitors first expand in the anteroposterior direction through a limited number of cell divisions and then, in virtual absence of cell proliferation, they stretch (Cechmanek and McFarlane, 2017; Kwan et al., 2012; Li et al., 2000; Moreno-Mármol et al., 2021) by strongly reducing their apico-basal axis while undergoing around an eightfold increase in their apical cell surface area (Moreno-Mármol et al., 2021). This transition from a pseudostratified to a squamous epithelium (Fig. 2A) occurs as RPE progenitors become fully specified with the onset of GRNs that make RPE cells rapidly molecularly diverge from their progenitors (Buono et al., 2021). Consistent with their squamous appearance, RPE cells begin to express keratins, connexins and desmosomal proteins that, in a speculative view, may confer a particular mechanical strength and a ‘syncytial-like’ behaviour to this tissue (Moreno-Mármol et al., 2021). These molecular changes, together with an important cytoskeletal reorganization, sustain RPE cell stretching (Moreno-Mármol et al., 2018; Moreno-Mármol et al., 2021). Indeed, inhibition of myosin II in a few RPE cells, or interference with microtubule depolymerization in RPE progenitors, prevents both cell flattening and OV folding, whereas similar focal perturbations in the NR impact on OV convexity but not in RPE flattening, suggesting a cell-autonomous control. Thus, cell-autonomous RPE stretching is a rather powerful mechanical force for teleost OC formation given that compromised stretching in only a few cells is sufficient to decrease OV folding (Moreno-Mármol et al., 2021). In summary, morphogenesis of the teleost OC depends on the tension generated by the coordinated shape changes that the NR and the RPE undergo, with the additional contribution of cell involution from the inner to outer layer of the OV (Fig. 2A).

An unresolved issue is whether and how these morphogenetic forces are coordinated and sensed. Part of the coordination may rely on inner-outer layer interactions, mediated by molecules such as semaphorins and their plexin receptors, as abrogation of their expression results in incomplete ventral OC formation (Cechmanek et al., 2021). It is tempting to speculate that mechano-sensor and -transducer proteins, such as Yes1-associated transcriptional regulator 1 (Yap1), may instead be involved in sensing RPE tension, a function that Yap1 exerts in many different tissues (Totaro et al., 2018). This possibility is supported by the observation that, in zebrafish *yap1* mutants, the RPE develops with a patchy appearance (Miesfeld et al., 2015), suggestive of failed cellular interaction. Furthermore, heterozygous loss-of-function mutations in YAP1 have been found in individuals affected by coloboma (DeYoung et al., 2022; Williamson and FitzPatrick, 2014), a defect in which the OF does not seal up (discussed in more detail later). Nevertheless, it should be noted that Yap1 also acts as a co-factor for the Tead family of transcription factors (TFs), which are downstream effectors of Hippo signalling and are among the first TFs to be recruited during RPE specification (Buono et al., 2021). Accordingly, RPE cells are completely absent in *yap-taz* zebrafish double mutants (*taz* being a *yap* paralog), indicating that the Tead/Taz/Yap1 complex is involved in RPE specification (Miesfeld et al., 2015). This is also supported by the observation that, in *Yap1* mouse mutants, the RPE acquires NR characteristics (Kim et al., 2016). Furthermore, different studies have demonstrated that Yap1 participates in the regulation of RPE, NR and CM proliferation at later stages of mouse eye development (Moon et al., 2018; Sun et al., 2020). It might, therefore, be difficult to distinguish the potential mechano-sensing activities of Yap1 in the RPE from these other functions.

## Folding of the optic vesicle in species with a longer embryonic developmental time

In contrast to teleosts, the transition of the amniote OV into an OC takes place in the order of days, with a considerable variability according to the gestational period of the species (Fig. 1B), so that human OV folding is much slower than that of the zebrafish (Fig. 1B). The morphology of the OV is also different, with a balloon shape constituted by a pseudostratified epithelium everted from the neural tube (Fig. 2B). Its dorsal region differentiates as RPE, the intermediate region as NR, whereas the optic stalk originates from the most ventral region (Box 2) (Martinez-Morales et al., 2017). The NR/RPE hinge regions become apparent as the NR bends inwards (Figs 1A and 2B).

Initial descriptions on how this ‘balloon’ folds came mostly from the analysis of static images from chick, mouse and even human embryos (Coulombre, 1969; Hilfer, 1983; O'Rahilly and Müller, 2010). These studies supported the ‘induction’ hypothesis, proposed by Hans Spemann, according to which the interaction between the OV neuroepithelium and the overlying surface ectoderm was required to form the OC. The nearing of the OV to the ectoderm was thought to induce ectoderm thickening to form the lens placode (see Glossary, Box 1), which, in turn, triggered OV folding (Chow and Lang, 2001). This idea was further supported by the observation of cytoplasmic extensions connecting the two tissues in different species, including humans (Chauhan et al., 2009; Mann, 1928; McAvoy, 1980), and by a number of genetic studies. For example, specific inactivation of *Sox2* and *Pax6* (two TFs belonging to the eye GRNs; Beccari et al., 2013) in the mouse lens ectoderm (see Glossary, Box 1) not only prevents lens placode formation but also OV folding (Smith et al., 2009), supporting a role for the lens ectoderm in OC morphogenesis.

This long-lasting belief started to be questioned with the generation of the first OC organoids derived from mouse ESCs (Eiraku et al., 2011). This seminal study showed that mouse ESCs (mESCs) can acquire eye field identity and then develop into an OC through an intrinsic self-organizing program, independently of the presence of a lens ectoderm/placode (Eiraku et al., 2011). This self-organization was subsequently reproduced using human ESCs (Lowe et al., 2016; Mellough et al., 2015; Nakano et al., 2012) and iPSCs (Gabriel et al., 2021; Meyer et al., 2011), and it is perhaps further supported by the histological analysis of the eye of individuals with congenital primary aphakia, a rare disorder in which the lens is missing but the OC still forms (Manschot, 1963; Valleix et al., 2006). Nevertheless, the low and variable frequency with which properly folded OCs form, especially when originating from human ESC or iPSCs (Capowski et al., 2019; Nakano et al., 2012), improves in culture conditions in which additional forebrain, primordial lens- and cornea-like structures also develop (Gabriel et al., 2021). There is also little understanding of the cell type identities associated with OV organoids that support their integrity (Decembrini et al., 2014) or of the composition of the surrounding ECM, which may have an important role in the successful generation of OCs. In fact, the ECM seems to promote early NR invagination in chick embryos (Oltean et al., 2016). In all, the proposed tissue interactions between the OV and the lens ectoderm/placode (Chow and Lang, 2001; Graw, 2003) may still have, at least, the role of optimizing OV folding into a proper cup and/or influencing OV elongation. According to computational models, this elongation seems to be needed to optimize OV invagination (Hosseini and Taber, 2018).

Independently of the aforementioned open questions, live imaging of *in vitro* OC generation has shown that neuroepithelial cells undergo a stepwise and domain-specific (NR versus hinge versus RPE) transformation of their morphology (Fig. 2), with tissue-autonomous capacity to generate and interpret mechanical forces (Eiraku et al., 2011, 2012; Okuda et al., 2018). Careful analysis of these changes, combined with computational modelling, has generated the so called ‘relaxation-expansion’ model of *in vitro* mammalian OC formation (Eiraku et al., 2011, 2012; Okuda et al., 2018). According to the model, the OV folds in different phases that involve changes in individual cell morphology (e.g. apical or basal surface contraction, cell flattening, etc.), in physical properties (stiffening and softening) and in adhesion (to the surrounding cells and ECM), together with cell division and positional rearrangements.

ESCs or iPSCs, cultured in media formulated for generating OCs, initially form spherical vesicles, some of which begin to acquire NR and RPE characteristics. Two main changes drive the folding of the sphere – the cell-autonomous convex invagination of the NR and the constriction of hinge cells along their apico-basal length (Fig. 2B) (Okuda et al., 2018) – both of which require cytoskeletal rearrangements. While acquiring their fate, NR cells redistribute their actomyosin content with increased accumulation at the basolateral surfaces and a decrease at the apical side. According to several studies of epithelial sheet bending (Heisenberg and Bellaïche, 2013) and pharmacological interferences with actomyosin function (Eiraku et al., 2011; Lowe et al., 2016; Okuda et al., 2018), this actomyosin reorganization induces a differential basal constriction and apical relaxation in neuroepithelial cells, so that their end-feet are, respectively, stiffer and softer, promoting an autonomous inward bending of the tissue (Okuda et al., 2018). This bending is likely strengthened by a more prominent intracellular adhesion at the basal side and the interaction with the ECM (Boucherie et al., 2013; Lowe et al., 2016), as observed in zebrafish and chick embryos (Nicolas-Perez et al., 2016; Oltean et al., 2016). The inward bending of the NR layer, in turn, imposes a particular strain on hinge cells. In organoids, these cells assume a rather sharp wedge shape (Fig. 2B), owing to lateral constriction along the apico-basal axis mediated by strain-induced calcium transients (Okuda et al., 2018). This constriction mechanism further facilitates OV folding and likely explains part of the differences between the shape of NR and hinge cells. However, a substantial redistribution of actomyosin with an inverted apico-basal distribution must also occur, given that, in hinge cells, the apical end-feet are quite narrow (Fig. 2B). Whether these changes are the result of the sole NR-derived strain or whether the RPE also exerts mechanical forces on hinge cells, as proposed in fishes (Heermann et al., 2015; Moreno-Mármol et al., 2021), is unclear. However, in ESC-derived organoids, the generation of abutting NR and RPE is crucial for the differentiation of the CM (Kuwahara et al., 2015). Furthermore, in mouse mutants in which the RPE is not specified but acquires a NR identity (Hägglund et al., 2013; Martinez-Morales et al., 2001; Tang et al., 2010), wedge-shaped hinge cells are virtually absent and OV folding is strongly compromised (Martinez-Morales et al., 2001).

Additional studies support the relevance of RPE and CM identity acquisition for amniote OV folding. In mouse organoids, failed RPE specification prevents the initial steps of OV invagination (Eiraku et al., 2011), although, once the OC is formed, the RPE seems to function as a shell, with high mechanical rigidity and a strong actomyosin activation that, if broken, does not impact on the convex NR shape (Eiraku et al., 2011). OC malformations have also been reported after genetic manipulations of components of the Wnt and Fgf signalling pathways that interfere with the acquisition of RPE and CM identity and/or OV growth (Balasubramanian et al., 2021; Bankhead et al., 2015; Carpenter et al., 2015; Fuhrmann et al., 2022). For example, failed secretion of lens-derived Wnt ligands perturbs proliferation of the peripheral region of the eye cup, with a more evident effect on cells of the RPE (Carpenter et al., 2015). This defect is associated with a poorly folded, ‘saucer’ shaped OC (Balasubramanian et al., 2021; Carpenter et al., 2015). Similarly, preventing post-translational modifications, and thus secretion, of Wnt ligands in the eye field diminishes OV growth and impairs its folding (Fuhrmann et al., 2022). In other words, RPE specification (Balasubramanian et al., 2021; Hägglund et al., 2013; Martinez-Morales et al., 2001; Tang et al., 2010;) and growth (Carpenter et al., 2015; Fuhrmann et al., 2022) are prerequisites for OC generation *in vivo*.

More likely, the NR and RPE prospective layers need to maintain an adequate proportion to support OV folding: if one of the two layers is shorter or larger than the other, the vesicle does not invaginate (Balasubramanian et al., 2021; Carpenter et al., 2015; Moreno-Mármol et al., 2021; Okuda et al., 2018), perhaps because the generated tension is unbalanced.

## Differences in OC morphogenesis among vertebrate species

The need for a balanced proportion of NR and RPE holds true for both fast-developing teleosts and slow-developing amniotes. However, the mechanism by which these species achieve the right proportion of layers represents one of their salient morphogenetic differences. In teleosts, cell proliferation is dispensable for OC formation (Kwan et al., 2012) and its inhibition has no effect on RPE expansion during OV folding (Cechmanek and McFarlane, 2017). Rather, the RPE seems to cease mitotic division and differentiates simultaneously with OV folding (Buono et al., 2021; Moreno-Mármol et al., 2021). Proliferation also minimally contributes to the growth of the prospective zebrafish NR, which increases its surface through rim involution, well in line with the observation that in zebrafish the outer OV layer loses cells (from 587 to 432), while the inner layer gains them, in numbers that cannot be explained only by cell proliferation (Li et al., 2000). Thus, cell stretching and cell flow are the solutions adopted by fast-developing species to maintain an appropriate equilibrium between the two OV layers (Moreno-Mármol et al., 2021), which, in both teleosts and amniotes, seems to be a prerequisite for OV folding (Carpenter et al., 2015; Moreno-Mármol et al., 2021; Okuda et al., 2018). Indeed, the 6 h duration of this process is far less than the estimated 10 h needed for a single cell cycle in the OV neuroepithelium (Li et al., 2000).

In species with slower development, rim involution has not so far been described, and perhaps rightly so, as the OV has the time to grow by cell division. This also explains the different RPE development in amniotes, in which cells do not undergo a marked stretching and keep dividing during OV folding, with a remarkable inverse correlation between proliferation rate and apico-basal length among species (Moreno-Mármol et al., 2021). In humans, the OC RPE layer is composed of a highly mitotic pseudostratified epithelium that barely differs from the NR layer (Moreno-Mármol et al., 2021), in striking contrast to the zebrafish OC morphology (Fig. 2C,D). Thus, in humans and in mammals more broadly, the RPE acquires a final cuboidal, not squamous, appearance at a slower pace, with a step-wise differentiation process, as recently supported by RNA-seq analysis of human OC (Hu et al., 2019).

Other differences in OV morphogenesis between species relate to the species-specific organ size and intrinsic developmental clocks. This is evident, for example, when comparing the development of eye organoids from mESCs and human ESCs (hESCs). OC derived from human cells requires a longer generation time, as occurs during embryonic development (Fig. 1B). This internal clock is also present in teleosts, as also shown by the isochronic transplantation of zebrafish blastomeres into a medaka host, where they develop into a NR according to their faster intrinsic dynamics (Fuhrmann et al., 2020). Similarly, hESC-derived OC are consistently larger (about twofold) and the NR layer is twice as thick as that obtained from mouse cells, reflecting the relative differences in the respective embryos at equivalent stages (Nakano et al., 2012). The human NR layer seems also to retain an intrinsic tendency to become apically convex, if isolated from the rest of the organoid after initial invagination, a behaviour not observed with mouse cells (Eiraku et al., 2011; Nakano et al., 2012). This difference perhaps depends on a distinct composition of the basal membrane or is favoured by a more apical localization of the nuclei in the human cells that forces apical expansion (Nakano et al., 2012). This observation is intriguing, especially considering that the large majority of human iPSC eye organoids develop as retinal spheres with an ectopic RPE patch (O'Hara-Wright and Gonzalez-Cordero, 2020). Although culture conditions might be, in part, responsible for this difference, it will be interesting to determine what the underlying molecular differences between human iPSC- and ESC-derived OC organoids are, as this may provide additional insights on eye morphogenesis.

Evolutionary developmental comparisons of the morphogenetic events that shape the eye primordium have just begun and with the large variability of existing camera eyes in the animal kingdom (Koenig and Gross, 2020), we envisage that additional variations will be discovered, especially when analysing non-conventional model species. In amniotes, apoptosis has a relevant role in refining eye morphogenesis, with massive apoptotic death in the developing lens, OF fusion and differentiating retina (Frade et al., 1997; Trousse et al., 2001). However, its potential participation in OV folding has not being specifically addressed, although mutations in genes involved in apoptosis are among the genetic causes of congenital eye defects [e.g. holocytochrome C synthase (HCCS); Indrieri et al., 2013]. The potential relevance of apoptosis in morphogenesis is further supported by the evidence that, during their extrusion from the neural tube epithelium, apoptotic cells generate forces that contribute to the acquisition of the neural tube conformation (Roellig et al., 2022). Similarly, this mechanism could be particularly important to shape the tubular eyes present in some deep-sea fishes (de Busserolles et al., 2020) or other environmental adaptations of the camera eye. These, in turn, might provide hints for a better understanding of congenital human eye malformations.

## Understanding inborn eye malformations through vertebrate environmental adaptations

**Fig. 3.**
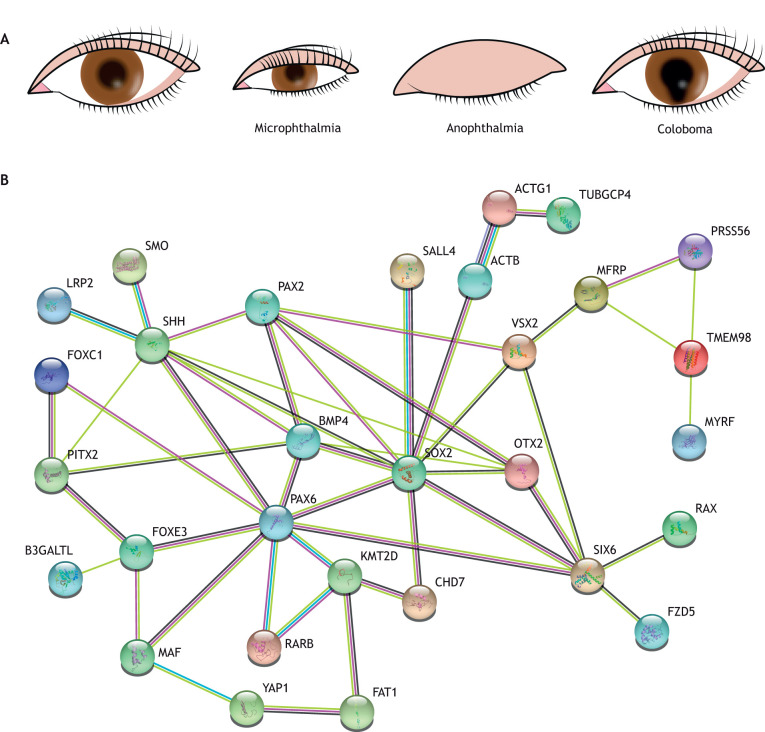
**Inborn eye malformations and possible interactions among causative defective proteins.** (A) Schematic representation of the eye in individuals affected by microphthalmia, anophthalmia or ventral coloboma. (B) STRING analysis of proteins encoded by genes responsible for microphthalmia, anophthalmia and coloboma (MAC). Many proteins are potentially functionally connected, with SOX2 acting as the principal node.

Microphthalmia, anophthalmia and coloboma (MAC) is a spectrum of rare conditions (the estimated prevalence is 2-14/100,000 births), in which the eye is either reduced in size (microphthalmia) or virtually absent (anophthalmia). Coloboma instead derives from the poor growth of any of the tissues that compose the anterior segment of the eye and/or the retina/optic stalk or from alterations in the molecular components that enable OF fusion (Fig. 1A and 3A) (Plaisancié et al., 2019; Williamson and FitzPatrick, 2014). MAC can exist in isolation or, more frequently, as part of complex syndromes. These malformations are thought to arise from abnormal eye morphogenesis and more than half of the cases already have a molecular diagnosis (Slavotinek, 2019; Williamson and FitzPatrick, 2014). However, the precise defective morphogenetic events responsible for these malformations are still unclear (Plaisancié et al., 2019). *De novo* or inherited heterozygous mutations in the human *SOX2* or *OTX2* loci are the most frequently identified genetic causes of MAC, with a phenotypic severity that can vary even among family members (Plaisancié et al., 2019). *SOX2* and *OTX2* have a crucial role in the specification of the entire and anterior neural plate, respectively (Acampora et al., 2001; Pevny and Nicolis, 2010), but they have been shown to co-regulate the expression of RAX (Danno et al., 2008), a TF that is required for the initial evagination of the OV (Mathers et al., 1997; Rembold et al., 2006). Indeed, their haploinsufficiency seems to affect mostly the eye, perhaps because the specification of the retinal, telencephalic and hypothalamic precursors seems to require different activity levels of these TFs (Beccari et al., 2012; Bernard et al., 2014; Martinez-Morales et al., 2001). Other and less frequent mutations, continuously updated in a MAC gene database (https://panelapp.genomicsengland.co.uk/panels/509/), have been found in genes belonging to GRNs that specify eye tissues, including TFs and members of signalling pathways (Table 2). Other mutations are instead in effector genes involved in cell adhesion, cytoskeletal rearrangements and mechano-sensing, or ECM components (Table 2), all molecular classes that have been implicated in OC morphogenesis. Notably, enrichment analysis of possible protein-protein interactions using the STRING software (https://string-db.org/) shows that many of the proteins encoded by MAC-responsible genes are potentially functionally connected, with SOX2 acting as principal node (Fig. 3B). Recent studies have also shown that Sox2 can recruit distant elements that are necessary for the expression of many forebrain genes, including those involved in eye specification (Bertolini et al., 2019; D'Aurizio et al., 2022). Furthermore, several of these identified putative SOX2-regulated enhancers are linked to MAC-responsible genes, including some of the above-mentioned loci or other factors either associated with related eye defects (e.g. nuclear factor erythroid 2–related factor 2 (NR2F); Bertacchi et al., 2019) or proposed as MAC candidates, such as myeloid ecotropic viral integration site 1 (MEIS1) (Marcos et al., 2015), cell adhesion-associated, oncogene regulated (CDON) (Cardozo et al., 2014; Reis et al., 2020; Zhang et al., 2009) and ventral anterior homeobox 1 (VAX1) (Slavotinek et al., 2012).

**Table 2.**
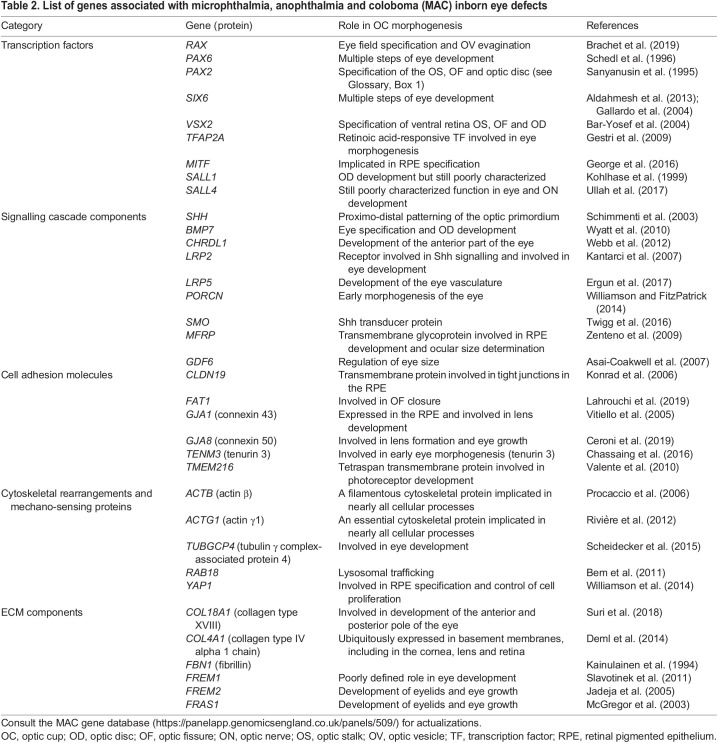
List of genes associated with microphthalmia, anophthalmia and coloboma (MAC) inborn eye defects

Taking these observations together, it is tempting to speculate that the insufficient level of SOX2 or OTX2 expression levels in individuals with MAC, particularly in those with anophthalmia and microphthalmia, may arise by a reduced recruitment of progenitors into the eye field and/or their poor specification as eye tissue. Eye field defects might be followed by an imbalance between the two OV layers, thereby destabilizing OC morphogenesis and its related cellular reorganizations. These initial destabilizations might be further reinforced by potentially lower levels of expression of a wide variety of SOX2- (Bertolini et al., 2019) and OTX2- (Sakai et al., 2017) regulated genes, many of which are similarly involved in morphogenetic events. Mutations in genes functionally linked to SOX2 and/or OTX2, or to their downstream effectors or to variations in regulatory elements, to which these TFs might bind, could have similar effects in destabilizing OV/OC morphogenesis and growth, eventually leading to its regression. However, no studies so far have directly addressed these possibilities. Indeed, the large number of available mouse mutants with MAC-resembling phenotypes have not yet been analysed with a specific focus on OC formation. Furthermore, most of the studies taking advantage of human retinal organoids have been directed towards solving disorders that cause blindness, such as retinitis pigmentosa or age-related macular degeneration (Gagliardi et al., 2019). Therefore, a detailed analysis of how MAC arises awaits further investigation.

Additional hints on how MAC phenotypes may develop come from the study of vertebrate species that are blind as a result of evolutionary adaptations to extreme environments, such as cave fish or terrestrial subterranean animals, such as the mole. Among the many teleosts that have undergone environmental adaptations (Ravi and Venkatesh, 2008), the *Astyanax mexicanus* (the Mexican tetra, or ‘blind cave fish’) has emerged as an attractive model for studying the development of a colobomatous, microphthalmic eye and its regression. *Astyanax mexicanus* exists as a river-dwelling surface form and a blind cave-dwelling morph. The adult cave morph has no eyes, although its embryos develop well-formed but small OCs that then regress in a stereotypical manner (Jeffery, 2020). Detailed comparison of the surface and cave early embryos shows that the cavefish begins to neurulate slightly earlier and has a smaller eye field than the corresponding surface morph (Agnès et al., 2022). This reduction has been associated with an earlier and more-expanded expression of Shh and Fgf8 (Pottin et al., 2011; Yamamoto et al., 2004), two morphogens that contribute to anterior neural plate patterning. This smaller eye field has less precise molecular borders with the adjacent telencephalic and hypothalamic fields, and shows decreased expression levels of *rx3* (an ortholog of the mammalian *Rax* gene), one of the TFs that confers eye identity and enables OV evagination (Rembold et al., 2006). Reduced *rx3* expression, together with DNA hypermethylation of other eye-specific genes (Gore et al., 2018), might explain why the cavefish OV evagination and elongation are somewhat abnormal and proceed for longer than they do in the surface form (Devos et al., 2021). As in the surface form, basal constriction of the cavefish retinal progenitors induces the initial bending of the OV but folding does not progress efficiently so that the OC remains rather flat (Devos et al., 2021), resembling the *ojoplano* phenotype (Martinez-Morales et al., 2009). Abnormal rim involution and RPE flattening, together with lens alterations, are the likely causes of these abnormal shapes that further lead to an evident coloboma (Devos et al., 2021).

In parallel, SOX2 haploinsufficiency may cause poor activation of RAX with consequences similar to those observed in *Astyanax*. In support of this possibility, a recent study points to the compromised activity of a distant enhancer as an explanation for the poor and fuzzy *rx3* expression in the *Astyanax* cave morph (Leclercq et al., 2022 preprint). Furthermore, *Sox2* seems to directly activate Rax/Rx3 expression in different species (Beccari et al., 2012). Accelerated changes in ocular-specific transcriptional enhancers have been also shown to occur in the genome of different types of moles (Partha et al., 2017), with genes involved in lens and retina development being the most affected. Thus, variants in regulatory elements controlling the expression of genes belonging to eye GRNs may likely explain at least part of the significant number of MAC cases that remain molecularly undiagnosed.

## Conclusions and perspectives

The acquisition of a proper 3D shape is crucial for the correct function of the eye in human and other vertebrate species. Any deviation from the expected eye geometry leads to poor or even absent vision. Therefore, understanding how the eye tissues develop and rearrange to generate a final cup-shaped camera eye may help to find strategies for solving visual defects. There have been many advances in our basic knowledge of how the eye acquires its shape early in development, mostly enabled using transparent vertebrate embryos and the increasing progress in mammalian organoid technology. These are accessible models amenable to genetic, pharmacological, surgical and mechanical manipulations that, together with mathematical and physical models, have identified the motors of OC formation.

Yet there are several unanswered questions that need to be addressed, such as the puzzling interdependence between lens and neural tissue. The lens tissue is no doubt a source of factors that influence OV development and specification, but does it also exert mechanical forces that help OV folding? Related to these questions, a recent study has reported that conditional deletion of *Arl13b*, a gene required for ciliogenesis in the mouse OV, disrupts morphogenesis so that the lens is abnormally surrounded by an inverted OC, in which the RPE layer faces the surface (Fiore et al., 2020). The primary cilium is an important cellular sensing structure and mutations in genes encoding its structural components are among identified causes of MAC (Plaisancié et al., 2019; Williamson and FitzPatrick, 2014). The *Arl13b* mutant phenotype seems to be linked to abnormal sensing of Shh signalling, but how this inverted shape arises is unclear and indicates an important contribution of the primary cilium to eye morphogenesis. When does the cilium form in the progenitors? Is it important only in a subset of OV progenitors? Does it contribute to sense cell tension and how?

Other highly relevant questions relate to the contribution of the surrounding mesenchyme and cephalic neural crest cells to OC formation. Although there is related information, these questions have not been systematically addressed. A recent interesting study has shown that the extensive evagination and invagination movements of the zebrafish eye exert traction forces on the adjacent olfactory placode, influencing its development (Monnot et al., 2022). Does the opposite hold true? Does this influence exist in other species in which the two structures are located further apart? Many of these questions could be addressed by studying early eye morphogenesis in species that are not currently raised in laboratory conditions. In this sense, the use of unconventional animal models such as *Astyanax*, or fish with other environmental adaptations, such as fish with tubular eyes (which have a main and an accessory retina), may help explain different aspects of morphogenesis and their underlying motors.

Very recent technological improvements use mESCs to grow *ex utero* post-gastrulation synthetic whole embryos (sEmbryos) that can reach a development stage comparable with embryonic day (E)8.5 embryos (Amadei et al., 2022; Tarazi et al., 2022). Although this stage has limited usefulness for studying eye-field specification, future advances may enable further development of sEmbryos and thereby facilitate *ex utero* manipulations and imaging of the mammalian eye, furthering our understanding of its morphogenesis. Furthermore, the generation of OC organoids from iPSCs derived from individuals with MAC will allow their 3D development to be followed in real time. For example, generating such models from individuals carrying *SOX2* or *OTX2* mutations will certainly be worthwhile. Furthermore, improving the development of human iPSC-derived OC organoids may in itself represent an important opportunity for learning more about how the eye forms. Indeed, in many cases the RPE develops as a clumped tissue attached to one side of the NR, failing to enwrap it. Investigating why it develops in this way might reveal more about the relationship between NR and RPE in OC formation. Lens, neural and mesenchymal ‘assembloids’ are also expected as future developments that should further clarify how the eye forms.

Many of the questions raised here can be addressed across species, with the possibility of comparing the developmental program of species-specific eye organoids as has been achieved for other organoids, including the possibility of generating inter-species assembloids. All these approaches may ultimately enhance our understanding of human eye morphogenesis and how MAC arises in humans; we predict that the field of eye morphogenesis and related malformations has exciting times ahead.

## References

[DEV200399C1] Acampora, D., Gulisano, M., Broccoli, V. and Simeone, A. (2001). Otx genes in brain morphogenesis. *Prog. Neurobiol.* 64, 69-95. 10.1016/S0301-0082(00)00042-3 11250063

[DEV200399C2] Agnès, F., Torres-Paz, J., Michel, P. and Rétaux, S. (2022). A 3D molecular map of the cavefish neural plate illuminates eye-field organization and its borders in vertebrates. *Development* 149, dev199966. 10.1242/dev.199966 35388410

[DEV200399C3] Aldahmesh, M. A., Khan, A. O., Hijazi, H. and Alkuraya, F. S. (2013). Homozygous truncation of SIX6 causes complex microphthalmia in humans. *Clin. Genet.* 84, 198-199. 10.1111/cge.12046 23167593

[DEV200399C4] Amadei, G., Handford, C. E., Qiu, C., De Jonghe, J., Greenfeld, H., Tran, M., Martin, B. K., Chen, D.-Y., Aguilera-Castrejon, A., Hanna, J. H. et al. (2022). Synthetic embryos complete gastrulation to neurulation and organogenesis. *Nature* 610, 143-153. 10.1038/s41586-022-05246-3 36007540 PMC9534772

[DEV200399C5] Arendt, D. (2003). Evolution of eyes and photoreceptor cell types. *Int. J. Dev. Biol.* 47, 563-571. 14756332

[DEV200399C6] Asai-Coakwell, M., French, C. R., Berry, K. M., Ye, M., Koss, R., Somerville, M., Mueller, R., Van Heyningen, V., Waskiewicz, A. J. and Lehmann, O. J. (2007). GDF6, a novel locus for a spectrum of ocular developmental anomalies. *Am. J. Hum. Genet.* 80, 306-315. 10.1086/511280 17236135 PMC1785352

[DEV200399C7] Baden, T. (2020). Vertebrate vision: Lessons from non-model species. *Semin. Cell Dev. Biol.* 106, 1-4. 10.1016/j.semcdb.2020.05.028 32532616

[DEV200399C8] Balasubramanian, R., Min, X., Quinn, P. M. J., Giudice, Q. L., Tao, C., Polanco, K., Makrides, N., Peregrin, J., Bouaziz, M., Mao, Y. et al. (2021). Phase transition specified by a binary code patterns the vertebrate eye cup. *Sci. Adv.* 7, eabj9846. 10.1126/sciadv.abj9846 34757798 PMC8580326

[DEV200399C9] Bankhead, E. J., Colasanto, M. P., Dyorich, K. M., Jamrich, M., Murtaugh, L. C. and Fuhrmann, S. (2015). Multiple requirements of the focal dermal hypoplasia gene porcupine during ocular morphogenesis. *Am. J. Pathol.* 185, 197-213. 10.1016/j.ajpath.2014.09.002 25451153 PMC4278246

[DEV200399C10] Bar-Yosef, U., Abuelaish, I., Harel, T., Hendler, N., Ofir, R. and Birk, O. S. (2004). CHX10 mutations cause non-syndromic microphthalmia/ anophthalmia in Arab and Jewish kindreds. *Hum. Genet.* 115, 302-309. 10.1007/s00439-004-1154-2 15257456

[DEV200399C11] Beccari, L., Conte, I., Cisneros, E. and Bovolenta, P. (2012). Sox2-mediated differential activation of Six3.2 contributes to forebrain patterning. *Development* 139, 151-164. 10.1242/dev.067660 22096077

[DEV200399C12] Beccari, L., Marco-Ferreres, R. and Bovolenta, P. (2013). The logic of gene regulatory networks in early vertebrate forebrain patterning. *Mech. Dev.* 130, 95-111. 10.1016/j.mod.2012.10.004 23111324

[DEV200399C13] Bem, D., Yoshimura, S.-I., Nunes-Bastos, R., Bond, F. F., Kurian, M. A., Rahman, F., Handley, M. T. W., Hadzhiev, Y., Masood, I., Straatman-Iwanowska, A. et al. (2011). Loss-of-function mutations in RAB18 cause warburg micro syndrome. *Am. J. Hum. Genet.* 88, 499-507. 10.1016/j.ajhg.2011.03.012 21473985 PMC3071920

[DEV200399C14] Bernard, C., Kim, H.-T., Torero Ibad, R., Lee, E. J., Simonutti, M., Picaud, S., Acampora, D., Simeone, A., Di Nardo, A. A., Prochiantz, A. et al. (2014). Graded Otx2 activities demonstrate dose-sensitive eye and retina phenotypes. *Hum. Mol. Genet.* 23, 1742-1753. 10.1093/hmg/ddt562 24234651

[DEV200399C15] Bertacchi, M., Gruart, A., Kaimakis, P., Allet, C., Serra, L., Giacobini, P., Delgado-Garcia, J. M., Bovolenta, P. and Studer, M. (2019). Mouse Nr2f1 haploinsufficiency unveils new pathological mechanisms of a human optic atrophy syndrome. *EMBO Mol. Med.* 11, e10291. 10.15252/emmm.201910291 31318166 PMC6685104

[DEV200399C16] Bertolini, J. A., Favaro, R., Zhu, Y., Pagin, M., Ngan, C. Y., Wong, C. H., Tjong, H., Vermunt, M. W., Martynoga, B., Barone, C. et al. (2019). Mapping the global chromatin connectivity network for Sox2 function in neural stem cell maintenance. *Cell Stem Cell* 24, 462-476.e6. 10.1016/j.stem.2019.02.004 30849367 PMC6506828

[DEV200399C17] Bogdanović, O., Delfino-Machín, M., Nicolás-Pérez, M., Gavilán, M. P., Gago-Rodrigues, I., Fernández-Miñán, A., Lillo, C., Ríos, R. M., Wittbrodt, J. and Martínez-Morales, J. R. (2012). Numb/Numbl-Opo antagonism controls retinal epithelium morphogenesis by regulating integrin endocytosis. *Dev. Cell* 23, 782-795. 10.1016/j.devcel.2012.09.004 23041384

[DEV200399C18] Boucherie, C., Mukherjee, S., Henckaerts, E., Thrasher, A. J., Sowden, J. C. and Ali, R. R. (2013). Brief report: self-organizing neuroepithelium from human pluripotent stem cells facilitates derivation of photoreceptors. *Stem Cells* 31, 408-414. 10.1002/stem.1268 23132794

[DEV200399C19] Brachet, C., Kozhemyakina, E. A., Boros, E., Heinrichs, C., Balikova, I., Soblet, J., Smits, G., Vilain, C. and Mathers, P. H. (2019). Truncating RAX mutations: anophthalmia, hypopituitarism, diabetes insipidus, and cleft palate in mice and men. *J. Clin. Endocrinol. Metab.* 104, 2925-2930. 10.1210/jc.2018-02316 30811539 PMC6543774

[DEV200399C20] Bryan, C. D., Chien, C.-B. and Kwan, K. M. (2016). Loss of laminin alpha 1 results in multiple structural defects and divergent effects on adhesion during vertebrate optic cup morphogenesis. *Dev. Biol.* 416, 324-337. 10.1016/j.ydbio.2016.06.025 27339294 PMC5288406

[DEV200399C21] Bryan, C. D., Casey, M. A., Pfeiffer, R. L., Jones, B. W. and Kwan, K. M. (2020). Optic cup morphogenesis requires neural crest-mediated basement membrane assembly. *Development* 147, dev181420. 10.1242/dev.181420 31988185 PMC7044464

[DEV200399C22] Buono, L., Corbacho, J., Naranjo, S., Almuedo-Castillo, M., Moreno-Marmol, T., De La Cerda, B., Sanabria-Reinoso, E., Polvillo, R., Díaz-Corrales, F. J., Bogdanovic, O. et al. (2021). Analysis of gene network bifurcation during optic cup morphogenesis in zebrafish. *Nat. Commun.* 12, 3866. 10.1038/s41467-021-24169-7 34162866 PMC8222258

[DEV200399C23] Capowski, E. E., Samimi, K., Mayerl, S. J., Phillips, M. J., Pinilla, I., Howden, S. E., Saha, J., Jansen, A. D., Edwards, K. L., Jager, L. D. et al. (2019). Reproducibility and staging of 3D human retinal organoids across multiple pluripotent stem cell lines. *Development* 146, dev171686. 10.1242/dev.171686 30567931 PMC6340149

[DEV200399C24] Cardozo, M. J., Sanchez-Arrones, L., Sandonis, A., Sanchez-Camacho, C., Gestri, G., Wilson, S. W., Guerrero, I. and Bovolenta, P. (2014). Cdon acts as a Hedgehog decoy receptor during proximal-distal patterning of the optic vesicle. *Nat. Commun.* 5, 4272. 10.1038/ncomms5272 25001599 PMC4102123

[DEV200399C25] Cardozo, M. J., Almuedo-Castillo, M. and Bovolenta, P. (2020). Patterning the vertebrate retina with morphogenetic signaling pathways. *Neuroscientist* 26, 185-196. 10.1177/1073858419874016 31509088

[DEV200399C26] Carpenter, A. C., Smith, A. N., Wagner, H., Cohen-Tayar, Y., Rao, S., Wallace, V., Ashery-Padan, R. and Lang, R. A. (2015). Wnt ligands from the embryonic surface ectoderm regulate “bimetallic strip” optic cup morphogenesis in mouse. *Development* 142, 972-982. 10.1242/dev.120022 25715397 PMC4352985

[DEV200399C27] Casares, F. and Almudi, I. (2016). Fast and furious 800. The retinal determination gene network in drosophila. In *Organogenetic Gene Networks: Genetic Control of Organ Formation* (ed. J. Castelli-Gair Hombría and P. Bovolenta), pp. 95-124. Cham: Springer International Publishing.

[DEV200399C28] Casey, M. A., Lusk, S. and Kwan, K. M. (2021). Build me up optic cup: Intrinsic and extrinsic mechanisms of vertebrate eye morphogenesis. *Dev. Biol.* 476, 128-136. 10.1016/j.ydbio.2021.03.023 33811855 PMC8848517

[DEV200399C29] Cavodeassi, F. (2018). Dynamic tissue rearrangements during vertebrate eye morphogenesis: insights from fish models. *J. Dev. Biol.* 6, 4. 10.3390/jdb6010004 29615553 PMC5875564

[DEV200399C30] Cechmanek, P. B. and Mcfarlane, S. (2017). Retinal pigment epithelium expansion around the neural retina occurs in two separate phases with distinct mechanisms. *Dev. Dyn.* 246, 598-609. 10.1002/dvdy.24525 28556369

[DEV200399C31] Cechmanek, P. B., Hehr, C. L. and Mcfarlane, S. (2021). Retinal pigment epithelium and neural retinal progenitors interact via Semaphorin 6D to facilitate optic cup morphogenesis. *eNeuro* 8, ENEURO.0053-21.2021. 10.1523/ENEURO.0053-21.2021 PMC8116109 33811086

[DEV200399C32] Ceroni, F., Aguilera-Garcia, D., Chassaing, N., Bax, D. A., Blanco-Kelly, F., Ramos, P., Tarilonte, M., Villaverde, C., Da Silva, L. R. J., Ballesta-Martã­Nez, M. J. et al. (2019). New GJA8 variants and phenotypes highlight its critical role in a broad spectrum of eye anomalies. *Hum. Genet.* 138, 1027-1042. 10.1007/s00439-018-1875-2 29464339

[DEV200399C33] Chassaing, N., Ragge, N., Plaisancié, J., Patat, O., Geneviève, D., Rivier, F., Malrieu-Eliaou, C., Hamel, C., Kaplan, J. and Calvas, P. (2016). Confirmation of TENM3 involvement in autosomal recessive colobomatous microphthalmia. *Am. J. Med. Genet. Part A* 170, 1895-1898. 10.1002/ajmg.a.37667 27103084

[DEV200399C34] Chauhan, B. K., Disanza, A., Choi, S.-Y., Faber, S. C., Lou, M., Beggs, H. E., Scita, G., Zheng, Y. and Lang, R. A. (2009). Cdc42- and IRSp53-dependent contractile filopodia tether presumptive lens and retina to coordinate epithelial invagination. *Development* 136, 3657-3667. 10.1242/dev.042242 19820184 PMC2761112

[DEV200399C35] Chen, Y.-C. and Desplan, C. (2020). Chapter Four - Gene regulatory networks during the development of the Drosophila visual system. In *Current Topics in Developmental Biology* (ed. I. S. Peter), pp. 89-125. Academic Press. 10.1016/bs.ctdb.2020.02.010 PMC7790576 32450970

[DEV200399C36] Chow, R. L. and Lang, R. A. (2001). Early Eye Development in Vertebrates. *Annu. Rev. Cell Dev. Biol.* 17, 255-296. 10.1146/annurev.cellbio.17.1.255 11687490

[DEV200399C37] Coulombre, A. J. (1969). Regulation of ocular morphogenesis. *Invest Ophthalmol* 8, 25-31. 5763845

[DEV200399C38] Danno, H., Michiue, T., Hitachi, K., Yukita, A., Ishiura, S. and Asashima, M. (2008). Molecular links among the causative genes for ocular malformation: Otx2 and Sox2 coregulate Rax expression. *Proc. Natl. Acad. Sci. USA* 105, 5408-5413. 10.1073/pnas.0710954105 18385377 PMC2291098

[DEV200399C39] D'Aurizio, R., Catona, O., Pitasi, M., Li, Y. E., Ren, B. and Nicolis, S. K. (2022). Bridging between mouse and human enhancer-promoter long-range interactions in neural stem cells, to understand enhancer function in neurodevelopmental disease. *Int. J. Mol. Sci.* 23, 7964. 10.3390/ijms23147964 35887306 PMC9322198

[DEV200399C40] De Busserolles, F., Fogg, L., Cortesi, F. and Marshall, J. (2020). The exceptional diversity of visual adaptations in deep-sea teleost fishes. *Semin. Cell Dev. Biol.* 106, 20-30. 10.1016/j.semcdb.2020.05.027 32536437

[DEV200399C41] Decembrini, S., Koch, U., Radtke, F., Moulin, A. and Arsenijevic, Y. (2014). Derivation of traceable and transplantable photoreceptors from mouse embryonic stem cells. *Stem Cell Reports* 2, 853-865. 10.1016/j.stemcr.2014.04.010 24936471 PMC4050344

[DEV200399C42] Deml, B., Reis, L. M., Maheshwari, M., Griffis, C., Bick, D. and Semina, E. V. (2014). Whole exome analysis identifies dominant COL4A1 mutations in patients with complex ocular phenotypes involving microphthalmia. *Clin. Genet.* 86, 475-481. 10.1111/cge.12379 24628545 PMC4163542

[DEV200399C43] Devos, L., Agnès, F., Edouard, J., Simon, V., Legendre, L., El Khallouki, N., Barbachou, S., Sohm, F. and Rétaux, S. (2021). Eye morphogenesis in the blind Mexican cavefish. *Biol. Open* 10, bio059031. 10.1242/bio.059031 34590124 PMC8565469

[DEV200399C44] Deyoung, C., Guan, B., Ullah, E., Blain, D., Hufnagel, R. B. and Brooks, B. P. (2022). De novo frameshift mutation in YAP1 associated with bilateral uveal coloboma and microphthalmia. *Ophthalmic Genet.* 43, 513-517. 10.1080/13816810.2022.2028299 35318877 PMC11610107

[DEV200399C45] Eiraku, M., Takata, N., Ishibashi, H., Kawada, M., Sakakura, E., Okuda, S., Sekiguchi, K., Adachi, T. and Sasai, Y. (2011). Self-organizing optic-cup morphogenesis in three-dimensional culture. *Nature* 472, 51-56. 10.1038/nature09941 21475194

[DEV200399C46] Eiraku, M., Adachi, T. and Sasai, Y. (2012). Relaxation-expansion model for self-driven retinal morphogenesis: a hypothesis from the perspective of biosystems dynamics at the multi-cellular level. *BioEssays* 34, 17-25. 10.1002/bies.201100070 22052700 PMC3266490

[DEV200399C47] Ergun, S. G., Akay, G. G., Ergun, M. A. and Perçin, E. F. (2017). LRP5-linked osteoporosis-pseudoglioma syndrome mimicking isolated microphthalmia. *Eur J. Med. Genetics* 60, 200-204. 10.1016/j.ejmg.2017.01.007 28111184

[DEV200399C48] Fiore, L., Takata, N., Acosta, S., Ma, W., Pandit, T., Oxendine, M. and Oliver, G. (2020). Optic vesicle morphogenesis requires primary cilia. *Dev. Biol.* 462, 119-128. 10.1016/j.ydbio.2020.02.016 32169553 PMC8167498

[DEV200399C49] Frade, J. M., Bovolenta, P., Martínez-Morales, J. R., Arribas, A., Barbas, J. A. and Rodríguez-Tébar, A. (1997). Control of early cell death by BDNF in the chick retina. *Development* 124, 3313-3320. 10.1242/dev.124.17.3313 9310326

[DEV200399C50] Fuhrmann, S. (2010). Eye morphogenesis and patterning of the optic vesicle. *Curr. Top. Dev. Biol.* 93, 61-84. 10.1016/B978-0-12-385044-7.00003-5 20959163 PMC2958684

[DEV200399C51] Fuhrmann, J. F., Buono, L., Adelmann, L., Martinez-Morales, J. R. and Centanin, L. (2020). Genetic developmental timing revealed by inter-species transplantations in fish. *Development* 147, dev192500. 10.1242/dev.192500 33033120

[DEV200399C52] Fuhrmann, S., Ramirez, S., Abouda, M. M. and Campbell, C. D. (2022). Porcn is essential for growth and invagination of the mammalian optic cup. *Front. Cell Dev. Biol.* 10, 1016182. 10.3389/fcell.2022.1016182 36393832 PMC9661423

[DEV200399C53] Gabriel, E., Albanna, W., Pasquini, G., Ramani, A., Josipovic, N., Mariappan, A., Schinzel, F., Karch, C. M., Bao, G., Gottardo, M. et al. (2021). Human brain organoids assemble functionally integrated bilateral optic vesicles. *Cell Stem Cell* 28, 1740-1757.e8. 10.1016/j.stem.2021.07.010 34407456

[DEV200399C54] Gagliardi, G., Ben M'barek, K. and Goureau, O. (2019). Photoreceptor cell replacement in macular degeneration and retinitis pigmentosa: A pluripotent stem cell-based approach. *Prog. Retin. Eye Res.* 71, 1-25. 10.1016/j.preteyeres.2019.03.001 30885665

[DEV200399C55] Gallardo, M. E., Rodríguez De Córdoba, S., Schneider, A. S., Dwyer, M. A., Ayuso, C. and Bovolenta, P. (2004). Analysis of the developmental SIX6 homeobox gene in patients with anophthalmia/ microphthalmia. *Am. J. Med. Genet. A* 129A, 92-94. 10.1002/ajmg.a.30126 15266624

[DEV200399C56] Gehring, W. J. (2014). The evolution of vision. *WIREs Dev. Biol.* 3, 1-40. 10.1002/wdev.96 24902832

[DEV200399C57] George, A., Zand, D. J., Hufnagel, R. B., Sharma, R., Sergeev, Y. V., Legare, J. M., Rice, G. M., Scott Schwoerer, J. A., Rius, M., Tetri, L. et al. (2016). Biallelic mutations in MITF cause coloboma, osteopetrosis, microphthalmia, macrocephaly, albinism, and deafness. *Am. J. Hum. Genet.* 99, 1388-1394. 10.1016/j.ajhg.2016.11.004 27889061 PMC5142105

[DEV200399C58] Gestri, G., Osborne, R. J., Wyatt, A. W., Gerrelli, D., Gribble, S., Stewart, H., Fryer, A., Bunyan, D. J., Prescott, K., Collin, J. R. O. et al. (2009). Reduced TFAP2A function causes variable optic fissure closure and retinal defects and sensitizes eye development to mutations in other morphogenetic regulators. *Hum. Genet.* 126, 791-803. 10.1007/s00439-009-0730-x 19685247 PMC3083835

[DEV200399C59] Gestri, G., Bazin-Lopez, N., Scholes, C. and Wilson, S. W. (2018). Cell behaviors during closure of the choroid fissure in the developing Eye. *Front. Cell Neurosci.* 12, 42. 10.3389/fncel.2018.00042 29515375 PMC5826230

[DEV200399C60] Giger, F. A. and Houart, C. (2018). The birth of the eye vesicle: when fate decision equals morphogenesis. *Front. Neurosci.* 12, 87. 10.3389/fnins.2018.00087 29515359 PMC5826324

[DEV200399C61] Goldsmith, T. H. (1990). Optimization, constraint, and history in the evolution of eyes. *Q. Rev. Biol.* 65, 281-322. 10.1086/416840 2146698

[DEV200399C62] Gore, A. V., Tomins, K. A., Iben, J., Ma, L., Castranova, D., Davis, A. E., Parkhurst, A., Jeffery, W. R. and Weinstein, B. M. (2018). An epigenetic mechanism for cavefish eye degeneration. *Nat. Ecol. Evol.* 2, 1155-1160. 10.1038/s41559-018-0569-4 29807993 PMC6023768

[DEV200399C63] Graw, J. (2003). The genetic and molecular basis of congenital eye defects. *Nat. Rev. Genet.* 4, 876-888. 10.1038/nrg1202 14634635

[DEV200399C64] Hägglund, A.-C., Berghard, A. and Carlsson, L. (2013). Canonical Wnt/β-catenin signalling is essential for optic cup formation. *PLoS ONE* 8, e81158. 10.1371/journal.pone.0081158 24324671 PMC3852023

[DEV200399C65] Heermann, S., Schütz, L., Lemke, S., Krieglstein, K. and Wittbrodt, J. (2015). Eye morphogenesis driven by epithelial flow into the optic cup facilitated by modulation of bone morphogenetic protein. *eLife* 4, e05216. 10.7554/eLife.05216 25719386 PMC4337729

[DEV200399C66] Heisenberg, C.-P. and Bellaïche, Y. (2013). Forces in tissue morphogenesis and patterning. *Cell* 153, 948-962. 10.1016/j.cell.2013.05.008 23706734

[DEV200399C67] Hilfer, S. R. (1983). Development of the eye of the chick embryo. *Scan Electron Microsc* 1353-1369. 6648345

[DEV200399C68] Hoshino, A., Ratnapriya, R., Brooks, M. J., Chaitankar, V., Wilken, M. S., Zhang, C., Starostik, M. R., Gieser, L., La Torre, A., Nishio, M. et al. (2017). Molecular anatomy of the developing human retina. *Dev. Cell* 43, 763-779.e4. 10.1016/j.devcel.2017.10.029 29233477 PMC5776731

[DEV200399C69] Hosseini, H. S. and Taber, L. A. (2018). How mechanical forces shape the developing eye. *Prog. Biophys. Mol. Biol.* 137, 25-36. 10.1016/j.pbiomolbio.2018.01.004 29432780 PMC6085168

[DEV200399C70] Hu, Y., Wang, X., Hu, B., Mao, Y., Chen, Y., Yan, L., Yong, J., Dong, J., Wei, Y., Wang, W. et al. (2019). Dissecting the transcriptome landscape of the human fetal neural retina and retinal pigment epithelium by single-cell RNA-seq analysis. *PLoS Biol.* 17, e3000365. 10.1371/journal.pbio.3000365 31269016 PMC6634428

[DEV200399C71] Indrieri, A., Conte, I., Chesi, G., Romano, A., Quartararo, J., Tatè, R., Ghezzi, D., Zeviani, M., Goffrini, P., Ferrero, I. et al. (2013). The impairment of HCCS leads to MLS syndrome by activating a non-canonical cell death pathway in the brain and eyes. *EMBO Mol Med* 5, 280-293. 10.1002/emmm.201201739 23239471 PMC3569643

[DEV200399C73] Jadeja, S., Smyth, I., Pitera, J. E., Taylor, M. S., Van Haelst, M., Bentley, E., Mcgregor, L., Hopkins, J., Chalepakis, G., Philip, N. et al. (2005). Identification of a new gene mutated in Fraser syndrome and mouse myelencephalic blebs. *Nat. Genet.* 37, 520-525. 10.1038/ng1549 15838507

[DEV200399C74] Jeffery, W. R. (2020). Astyanax surface and cave fish morphs. *Evodevo* 11, 14. 10.1186/s13227-020-00159-6 32676179 PMC7353729

[DEV200399C75] Kainulainen, K., Karttunen, L., Puhakka, L., Sakai, L. and Peltonen, L. (1994). Mutations in the fibrillin gene responsible for dominant ectopia lentis and neonatal Marfan syndrome. *Nat. Genet.* 6, 64-69. 10.1038/ng0194-64 8136837

[DEV200399C76] Kantarci, S., Al-Gazali, L., Hill, R. S., Donnai, D., Black, G. C. M., Bieth, E., Chassaing, N., Lacombe, D., Devriendt, K., Teebi, A. et al. (2007). Mutations in LRP2, which encodes the multiligand receptor megalin, cause Donnai-Barrow and facio-oculo-acoustico-renal syndromes. *Nat. Genet.* 39, 957-959. 10.1038/ng2063 17632512 PMC2891728

[DEV200399C77] Kim, J. Y., Park, R., Lee, J. H. J., Shin, J., Nickas, J., Kim, S. and Cho, S.-H. (2016). Yap is essential for retinal progenitor cell cycle progression and RPE cell fate acquisition in the developing mouse eye. *Dev. Biol.* 419, 336-347. 10.1016/j.ydbio.2016.09.001 27616714 PMC5125893

[DEV200399C78] Koenig, K. M. and Gross, J. M. (2020). Evolution and development of complex eyes: a celebration of diversity. *Development* 147, dev182923. 10.1242/dev.182923 33051250 PMC7578360

[DEV200399C79] Kohlhase, J., Taschner, P. E., Burfeind, P., Pasche, B., Newman, B., Blanck, C., Breuning, M. H., Ten Kate, L. P., Maaswinkel-Mooy, P., Mitulla, B. et al. (1999). Molecular analysis of SALL1 mutations in Townes-Brocks syndrome. *Am. J. Hum. Genet.* 64, 435-445. 10.1086/302238 9973281 PMC1377753

[DEV200399C80] Konrad, M., Schaller, A., Seelow, D., Pandey, A. V., Waldegger, S., Lesslauer, A., Vitzthum, H., Suzuki, Y., Luk, J. M., Becker, C. et al. (2006). Mutations in the tight-junction gene claudin 19 (CLDN19) are associated with renal magnesium wasting, renal failure, and severe ocular involvement. *Am. J. Hum. Genet.* 79, 949-957. 10.1086/508617 17033971 PMC1698561

[DEV200399C81] Kuwahara, A., Ozone, C., Nakano, T., Saito, K., Eiraku, M. and Sasai, Y. (2015). Generation of a ciliary margin-like stem cell niche from self-organizing human retinal tissue. *Nat. Commun.* 6, 6286. 10.1038/ncomms7286 25695148

[DEV200399C82] Kwan, K. M., Otsuna, H., Kidokoro, H., Carney, K. R., Saijoh, Y. and Chien, C.-B. (2012). A complex choreography of cell movements shapes the vertebrate eye. *Development* 139, 359-372. 10.1242/dev.071407 22186726 PMC3243097

[DEV200399C83] Lahrouchi, N., George, A., Ratbi, I., Schneider, R., Elalaoui, S. C., Moosa, S., Bharti, S., Sharma, R., Abu-Asab, M., Onojafe, F. et al. (2019). Homozygous frameshift mutations in FAT1 cause a syndrome characterized by colobomatous-microphthalmia, ptosis, nephropathy and syndactyly. *Nat. Commun.* 10, 1180. 10.1038/s41467-019-08547-w 30862798 PMC6414540

[DEV200399C85] Lamb, T. D., Collin, S. P. and Pugh, E. N. (2007). Evolution of the vertebrate eye: opsins, photoreceptors, retina and eye cup. *Nat. Rev. Neurosci.* 8, 960-976. 10.1038/nrn2283 18026166 PMC3143066

[DEV200399C86] Leclercq, J., Torres-Paz, J., Policarpo, M., Agnès, F. and Rétaux, S. (2022). Evolution of the regulation of developmental gene expression in blind Mexican cavefish. *bioRxiv*, 2022.07.12.499770. 10.1242/dev.202610 39007346

[DEV200399C87] Li, Z., Joseph, N. M. and Easter, S. S. (2000). The morphogenesis of the zebrafish eye, including a fate map of the optic vesicle. *Dev. Dyn.* 218, 175-188. 10.1002/(SICI)1097-0177(200005)218:1<175::AID-DVDY15>3.0.CO;2-K 10822269

[DEV200399C88] Lowe, A., Harris, R., Bhansali, P., Cvekl, A. and Liu, W. (2016). Intercellular adhesion-dependent cell survival and rock-regulated actomyosin-driven forces mediate self-formation of a retinal organoid. *Stem Cell Reports* 6, 743-756. 10.1016/j.stemcr.2016.03.011 27132890 PMC4939656

[DEV200399C89] Mann, I. C. (1928). *The Development of the Human Eye*. Oxford, England: Univ. Press.

[DEV200399C90] Manschot, W. A. (1963). Primary Congenital Aphakia. *Arch. Ophthalmol.* 69, 571-577. 10.1001/archopht.1963.00960040577007

[DEV200399C91] Marcos, S., Gonzalez-Lazaro, M., Beccari, L., Carramolino, L., Martin-Bermejo, M. J., Amarie, O., Mateos-San Martin, D., Torroja, C., Bogdanovic, O., Doohan, R. et al. (2015). Meis1 coordinates a network of genes implicated in eye development and microphthalmia. *Development* 142, 3009-3020. 10.1242/dev.122176 26253404

[DEV200399C92] Martinez-Morales, J. R. (2016). Vertebrate Eye Gene Regulatory Networks. In *Organogenetic Gene Networks: Genetic Control of Organ Formation* (ed. J. Castelli-Gair Hombría and P. Bovolenta), pp. 259-274. Cham: Springer International Publishing.

[DEV200399C93] Martinez-Morales, J. and Locascio, A. M. (2016). Vertebrate eye evolution. In *Organogenetic Gene Networks: Genetic Control of Organ Formation* (ed. J. Castelli-Gair Hombría and P. Bovolenta), pp. 275-298. Cham: Springer International Publishing.

[DEV200399C94] Martinez-Morales, J. R., Signore, M., Acampora, D., Simeone, A. and Bovolenta, P. (2001). Otx genes are required for tissue specification in the developing eye. *Development* 128, 2019-2030. 10.1242/dev.128.11.2019 11493524

[DEV200399C95] Martinez-Morales, J. R., Rembold, M., Greger, K., Simpson, J. C., Brown, K. E., Quiring, R., Pepperkok, R., Martin-Bermudo, M. D., Himmelbauer, H. and Wittbrodt, J. (2009). ojoplano-mediated basal constriction is essential for optic cup morphogenesis. *Development* 136, 2165-2175. 10.1242/dev.033563 19502481

[DEV200399C96] Martinez-Morales, J.-R., Cavodeassi, F. and Bovolenta, P. (2017). Coordinated morphogenetic mechanisms shape the vertebrate eye. *Front. Neurosci.* 11, 721. 10.3389/fnins.2017.00721 29326547 PMC5742352

[DEV200399C97] Mathers, P. H., Grinberg, A., Mahon, K. A. and Jamrich, M. (1997). The Rx homeobox gene is essential for vertebrate eye development. *Nature* 387, 603-607. 10.1038/42475 9177348

[DEV200399C98] Mcavoy, J. W. (1980). Cytoplasmic processes interconnect lens placode and optic vesicle during eye morphogenesis. *Exp. Eye Res.* 31, 527-534. 10.1016/S0014-4835(80)80011-X 7192638

[DEV200399C99] Mcgregor, L., Makela, V., Darling, S. M., Vrontou, S., Chalepakis, G., Roberts, C., Smart, N., Rutland, P., Prescott, N., Hopkins, J. et al. (2003). Fraser syndrome and mouse blebbed phenotype caused by mutations in FRAS1/Fras1 encoding a putative extracellular matrix protein. *Nat. Genet.* 34, 203-208. 10.1038/ng1142 12766769

[DEV200399C100] Mellough, C. B., Collin, J., Khazim, M., White, K., Sernagor, E., Steel, D. H. W. and Lako, M. (2015). IGF-1 Signaling Plays an Important Role in the Formation of Three-Dimensional Laminated Neural Retina and Other Ocular Structures From Human Embryonic Stem Cells. *Stem Cells* 33, 2416-2430. 10.1002/stem.2023 25827910 PMC4691326

[DEV200399C101] Meyer, J. S., Howden, S. E., Wallace, K. A., Verhoeven, A. D., Wright, L. S., Capowski, E. E., Pinilla, I., Martin, J. M., Tian, S., Stewart, R. et al. (2011). Optic vesicle-like structures derived from human pluripotent stem cells facilitate a customized approach to retinal disease treatment. *Stem Cells* 29, 1206-1218. 10.1002/stem.674 21678528 PMC3412675

[DEV200399C102] Miesfeld, J. B. and Brown, N. L. (2019). Chapter Ten - Eye organogenesis: A hierarchical view of ocular development. In *Current Topics in Developmental Biology* (ed. D. M. Wellik), pp. 351-393. Academic Press. 10.1016/bs.ctdb.2018.12.008 30797514

[DEV200399C103] Miesfeld, J. B., Gestri, G., Clark, B. S., Flinn, M. A., Poole, R. J., Bader, J. R., Besharse, J. C., Wilson, S. W. and Link, B. A. (2015). Yap and Taz regulate retinal pigment epithelial cell fate. *Development* 142, 3021-3032. 10.1242/dev.119008 26209646 PMC4582179

[DEV200399C104] Monnot, P., Gangatharan, G., Baraban, M., Pottin, K., Cabrera, M., Bonnet, I. and Breau, M. A. (2022). Intertissue mechanical interactions shape the olfactory circuit in zebrafish. *EMBO Rep.* 23, e52963. 10.15252/embr.202152963 34889034 PMC8811657

[DEV200399C105] Moon, K. H., Kim, H.-T., Lee, D., Rao, M. B., Levine, E. M., Lim, D.-S. and Kim, J. W. (2018). Differential expression of NF2 in neuroepithelial compartments is necessary for mammalian eye development. *Dev. Cell* 44, 13-28.e3. 10.1016/j.devcel.2017.11.011 29249622 PMC5760287

[DEV200399C106] Morcillo, J., Martínez-Morales, J. R., Trousse, F., Fermin, Y., Sowden, J. C. and Bovolenta, P. (2006). Proper patterning of the optic fissure requires the sequential activity of BMP7 and SHH. *Development* 133, 3179-3190. 10.1242/dev.02493 16854970

[DEV200399C107] Moreno-Mármol, T., Cavodeassi, F. and Bovolenta, P. (2018). Setting eyes on the retinal pigment epithelium. *Front. Cell Dev. Biol.* 6, 145. 10.3389/fcell.2018.00145 30406103 PMC6207792

[DEV200399C108] Moreno-Mármol, T., Ledesma-Terrón, M., Tabanera, N., Martin-Bermejo, M. J., Cardozo, M. J., Cavodeassi, F. and Bovolenta, P. (2021). Stretching of the retinal pigment epithelium contributes to zebrafish optic cup morphogenesis. *eLife* 10, e63396. 10.7554/eLife.63396 34545806 PMC8530511

[DEV200399C109] Nakano, T., Ando, S., Takata, N., Kawada, M., Muguruma, K., Sekiguchi, K., Saito, K., Yonemura, S., Eiraku, M. and Sasai, Y. (2012). Self-formation of optic cups and storable stratified neural retina from human ESCs. *Cell Stem Cell* 10, 771-785. 10.1016/j.stem.2012.05.009 22704518

[DEV200399C110] Nicolas-Perez, M., Kuchling, F., Letelier, J., Polvillo, R., Wittbrodt, J. and Martinez-Morales, J. R. (2016). Analysis of cellular behavior and cytoskeletal dynamics reveal a constriction mechanism driving optic cup morphogenesis. *Elife* 5, e15797. 10.7554/eLife.15797 27797321 PMC5110244

[DEV200399C111] O'hara-Wright, M. and Gonzalez-Cordero, A. (2020). Retinal organoids: a window into human retinal development. *Development* 147, dev189746. 10.1242/dev.189746 33361444 PMC7774906

[DEV200399C112] Okuda, S., Takata, N., Hasegawa, Y., Kawada, M., Inoue, Y., Adachi, T., Sasai, Y. and Eiraku, M. (2018). Strain-triggered mechanical feedback in self-organizing optic-cup morphogenesis. *Sci. Adv.* 4, eaau1354. 10.1126/sciadv.aau1354 30474058 PMC6248953

[DEV200399C113] Oltean, A., Huang, J., Beebe, D. C. and Taber, L. A. (2016). Tissue growth constrained by extracellular matrix drives invagination during optic cup morphogenesis. *Biomech. Model. Mechanobiol.* 15, 1405-1421. 10.1007/s10237-016-0771-8 26984743 PMC6014613

[DEV200399C114] O'Rahilly, R. and Müller, F. (2010). Developmental stages in human embryos: revised and new measurements. *Cells Tissues Organs (Print)* 192, 73-84. 10.1159/000289817 20185898

[DEV200399C115] Partha, R., Chauhan, B. K., Ferreira, Z., Robinson, J. D., Lathrop, K., Nischal, K. K., Chikina, M. and Clark, N. L. (2017). Subterranean mammals show convergent regression in ocular genes and enhancers, along with adaptation to tunneling. *eLife* 6, e25884. 10.7554/eLife.25884 29035697 PMC5643096

[DEV200399C116] Pevny, L. H. and Nicolis, S. K. (2010). Sox2 roles in neural stem cells. *Int. J. Biochem. Cell Biol.* 42, 421-424. 10.1016/j.biocel.2009.08.018 19733254

[DEV200399C117] Picker, A., Cavodeassi, F., Machate, A., Bernauer, S., Hans, S., Abe, G., Kawakami, K., Wilson, S. W. and Brand, M. (2009). Dynamic coupling of pattern formation and morphogenesis in the developing vertebrate retina. *PLoS Biol.* 7, e1000214. 10.1371/journal.pbio.1000214 19823566 PMC2751823

[DEV200399C118] Plaisancié, J., Ceroni, F., Holt, R., Zazo Seco, C., Calvas, P., Chassaing, N. and Ragge, N. K. (2019). Genetics of anophthalmia and microphthalmia. Part 1: Non-syndromic anophthalmia/microphthalmia. *Hum. Genet.* 138, 799-830. 10.1007/s00439-019-01977-y 30762128

[DEV200399C119] Pottin, K., Hinaux, H. and Rétaux, S. (2011). Restoring eye size in Astyanax mexicanus blind cavefish embryos through modulation of the Shh and Fgf8 forebrain organising centres. *Development* 138, 2467-2476. 10.1242/dev.054106 21610028

[DEV200399C120] Procaccio, V., Salazar, G., Ono, S., Styers, M. L., Gearing, M., Davila, A., Jimenez, R., Juncos, J., Gutekunst, C.-A., Meroni, G. et al. (2006). A mutation of β-actin that alters depolymerization dynamics is associated with autosomal dominant developmental malformations, deafness, and dystonia. *Am. J. Hum. Genet.* 78, 947-960. 10.1086/504271 16685646 PMC1474101

[DEV200399C121] Ravi, V. and Venkatesh, B. (2008). Rapidly evolving fish genomes and teleost diversity. *Curr. Opin. Genet. Dev.* 18, 544-550. 10.1016/j.gde.2008.11.001 19095434

[DEV200399C158] Reis, L. M., Basel, D., McCarrier, J., Weinberg, D. V. and Semina, E. V. (2020). Compound heterozygous splicing CDON variants result in isolated ocular coloboma. *Clin. Genet.* 98, 486-492. 10.1111/cge.13824 32729136 PMC8341436

[DEV200399C122] Rembold, M., Loosli, F., Adams, R. J. and Wittbrodt, J. (2006). Individual cell migration serves as the driving force for optic vesicle evagination. *Science* 313, 1130-1134. 10.1126/science.1127144 16931763

[DEV200399C123] Rivière, J.-B., Van Bon, B. W. M., Hoischen, A., Kholmanskikh, S. S., O'roak, B. J., Gilissen, C., Gijsen, S., Sullivan, C. T., Christian, S. L., Abdul-Rahman, O. A. et al. (2012). De novo mutations in the actin genes ACTB and ACTG1 cause Baraitser-Winter syndrome. *Nat. Genet.* 44, 440-444. 10.1038/ng.1091 22366783 PMC3677859

[DEV200399C124] Roellig, D., Theis, S., Proag, A., Allio, G., Bénazéraf, B., Gros, J. and Suzanne, M. (2022). Force-generating apoptotic cells orchestrate avian neural tube bending. *Dev. Cell* 57, 707-718.e6. 10.1016/j.devcel.2022.02.020 35303434 PMC8967407

[DEV200399C125] Sakai, A., Nakato, R., Ling, Y., Hou, X., Hara, N., Iijima, T., Yanagawa, Y., Kuwano, R., Okuda, S., Shirahige, K. et al. (2017). Genome-wide target analyses of Otx2 homeoprotein in postnatal cortex. *Front. Neurosci.* 11, 307. 10.3389/fnins.2017.00307 28620275 PMC5450002

[DEV200399C126] Sanyanusin, P., Schimmenti, L. A., Mcnoe, L. A., Ward, T. A., Pierpont, M. E., Sullivan, M. J., Dobyns, W. B. and Eccles, M. R. (1995). Mutation of the PAX2 gene in a family with optic nerve colobomas, renal anomalies and vesicoureteral reflux. *Nat. Genet.* 9, 358-364. 10.1038/ng0495-358 7795640

[DEV200399C127] Schedl, A., Ross, A., Lee, M., Engelkamp, D., Rashbass, P., Van Heyningen, V. and Hastie, N. D. (1996). Influence of PAX6 gene dosage on development: overexpression causes severe eye abnormalities. *Cell* 86, 71-82. 10.1016/S0092-8674(00)80078-1 8689689

[DEV200399C128] Scheidecker, S., Etard, C., Haren, L., Stoetzel, C., Hull, S., Arno, G., Plagnol, V., Drunat, S., Passemard, S., Toutain, A. et al. (2015). Mutations in TUBGCP4 alter microtubule organization via the γ-tubulin ring complex in autosomal-recessive microcephaly with chorioretinopathy. *Am. J. Hum. Genet.* 96, 666-674. 10.1016/j.ajhg.2015.02.011 25817018 PMC4385181

[DEV200399C129] Schimmenti, L. A., De La Cruz, J., Lewis, R. A., Karkera, J. D., Manligas, G. S., Roessler, E. and Muenke, M. (2003). Novel mutation in sonic hedgehog in non-syndromic colobomatous microphthalmia. *Am. J. Med. Genet. Part A* 116A, 215-221. 10.1002/ajmg.a.10884 12503095

[DEV200399C130] Sidhaye, J. and Norden, C. (2017). Concerted action of neuroepithelial basal shrinkage and active epithelial migration ensures efficient optic cup morphogenesis. *eLife* 6, e22689. 10.7554/eLife.22689 28372636 PMC5380436

[DEV200399C131] Sinn, R. and Wittbrodt, J. (2013). An eye on eye development. *Mech. Dev.* 130, 347-358. 10.1016/j.mod.2013.05.001 23684892

[DEV200399C133] Slavotinek, A. (2019). Genetics of anophthalmia and microphthalmia. Part 2: Syndromes associated with anophthalmia–microphthalmia. *Hum. Genet.* 138, 831-846. 10.1007/s00439-018-1949-1 30374660

[DEV200399C134] Slavotinek, A. M., Baranzini, S. E., Schanze, D., Labelle-Dumais, C., Short, K. M., Chao, R., Yahyavi, M., Bijlsma, E. K., Chu, C., Musone, S. et al. (2011). Manitoba-oculo-tricho-anal (MOTA) syndrome is caused by mutations in FREM1. *J. Med. Genet.* 48, 375-382. 10.1136/jmg.2011.089631 21507892 PMC4294942

[DEV200399C135] Slavotinek, A. M., Chao, R., Vacik, T., Yahyavi, M., Abouzeid, H., Bardakjian, T., Schneider, A., Shaw, G., Sherr, E. H., Lemke, G. et al. (2012). VAX1 mutation associated with microphthalmia, corpus callosum agenesis, and orofacial clefting: the first description of a VAX1 phenotype in humans. *Hum. Mutat.* 33, 364-368. 10.1002/humu.21658 22095910 PMC3401628

[DEV200399C136] Smith, A. N., Miller, L.-A., Radice, G., Ashery-Padan, R. and Lang, R. A. (2009). Stage-dependent modes of Pax6-Sox2 epistasis regulate lens development and eye morphogenesis. *Development* 136, 2977-2985. 10.1242/dev.037341 19666824 PMC2723069

[DEV200399C157] Soans, K. G., Ramos, A. P., Sidhaye, J., Krishna, A., Solomatina, A., Hoffmann, K. B., Schlüßler, R., Guck, J., Sbalzarini, I. F., Modes C. D. et al. (2022). Collective cell migration during optic cup formation features changing cell-matrix interactions linked to matrix topology. *Curr. Biol.* 32, 4817-4831.e9. 10.1016/j.cub.2022.09.034 36208624

[DEV200399C137] Sun, W. R., Ramirez, S., Spiller, K. E., Zhao, Y. and Fuhrmann, S. (2020). Nf2 fine-tunes proliferation and tissue alignment during closure of the optic fissure in the embryonic mouse eye. *Hum. Mol. Genet.* 29, 3373-3387. 10.1093/hmg/ddaa228 33075808 PMC7749708

[DEV200399C138] Suri, F., Yazdani, S., Chapi, M., Safari, I., Rasooli, P., Daftarian, N., Jafarinasab, M. R., Ghasemi Firouzabadi, S., Alehabib, E., Darvish, H. et al. (2018). COL18A1 is a candidate eye iridocorneal angle-closure gene in humans. *Hum. Mol. Genet.* 27, 3772-3786. 10.1093/hmg/ddy256 30007336

[DEV200399C139] Tang, K., Xie, X., Park, J.-I., Jamrich, M., Tsai, S. and Tsai, M.-J. (2010). COUP-TFs regulate eye development by controlling factors essential for optic vesicle morphogenesis. *Development* 137, 725-734. 10.1242/dev.040568 20147377 PMC2827684

[DEV200399C140] Tarazi, S., Aguilera-Castrejon, A., Joubran, C., Ghanem, N., Ashouokhi, S., Roncato, F., Wildschutz, E., Haddad, M., Oldak, B., Gomez-Cesar, E. et al. (2022). Post-gastrulation synthetic embryos generated ex utero from mouse naive ESCs. *Cell* 185, 3290-3306.e25. 10.1016/j.cell.2022.07.028 35988542 PMC9439721

[DEV200399C141] Totaro, A., Panciera, T. and Piccolo, S. (2018). YAP/TAZ upstream signals and downstream responses. *Nat. Cell Biol.* 20, 888-899. 10.1038/s41556-018-0142-z 30050119 PMC6186418

[DEV200399C142] Trousse, F., Esteve, P. and Bovolenta, P. (2001). Bmp4 mediates apoptotic cell death in the developing chick eye. *J. Neurosci.* 21, 1292-1301. 10.1523/JNEUROSCI.21-04-01292.2001 11160400 PMC6762245

[DEV200399C143] Twigg, S. R. F., Hufnagel, R. B., Miller, K. A., Zhou, Y., Mcgowan, S. J., Taylor, J., Craft, J., Taylor, J. C., Santoro, S. L., Huang, T. et al. (2016). A recurrent mosaic mutation in SMO, encoding the hedgehog signal transducer smoothened, is the major cause of curry-jones syndrome. *Am. J. Hum Genet.* 98, 1256-1265. 10.1016/j.ajhg.2016.04.007 27236920 PMC4908219

[DEV200399C160] Ullah, E., Wu, D., Madireddy, L., Lao, R., Ling-Fung Tang, P., Wan, E., Bardakjian, T., Kopinsky, S., Kwok, P.-Y., Schneider, A. et al. (2017). Two missense mutations in SALL4 in a patient with microphthalmia, coloboma, and optic nerve hypoplasia. *Ophthalmic Genet.* 38, 371-375. 10.1080/13816810.2016.1217550 27661448 PMC6238016

[DEV200399C144] Valente, E. M., Logan, C. V., Mougou-Zerelli, S., Lee, J. H., Silhavy, J. L., Brancati, F., Iannicelli, M., Travaglini, L., Romani, S., Illi, B. et al. (2010). Mutations in TMEM216 perturb ciliogenesis and cause Joubert, Meckel and related syndromes. *Nat. Genet.* 42, 619-625. 10.1038/ng.594 20512146 PMC2894012

[DEV200399C145] Valleix, S., Niel, F., Nedelec, B., Algros, M.-P., Schwartz, C., Delbosc, B., Delpech, M. and Kantelip, B. (2006). Homozygous nonsense mutation in the FOXE3 gene as a cause of congenital primary Aphakia in humans. *Am. J. Hum. Genet.* 79, 358-364. 10.1086/505654 16826526 PMC1559477

[DEV200399C146] Vallerga, S. (1994). The phylogenetic evolution of the visual system. In *Human and Machine Vision: Analogies and Divergencies* (ed. V. Cantoni), pp. 1-12. Boston, MA: Springer US.

[DEV200399C147] Vitiello, C., D'adamo, P., Gentile, F., Vingolo, E. M., Gasparini, P. and Banfi, S. (2005). A novel GJA1 mutation causes oculodentodigital dysplasia without syndactyly. *Am. J. Med. Gen. Part A* 133A, 58-60. 10.1002/ajmg.a.30554 15637728

[DEV200399C148] Webb, T. R., Matarin, M., Gardner, J. C., Kelberman, D., Hassan, H., Ang, W., Michaelides, M., Ruddle, J. B., Pennell, C. E., Yazar, S. et al. (2012). X-linked megalocornea caused by mutations in CHRDL1 identifies an essential role for ventroptin in anterior segment development. *Am. J. Hum. Genet.* 90, 247-259. 10.1016/j.ajhg.2011.12.019 22284829 PMC3276677

[DEV200399C149] Wyatt, A. W., Osborne, R. J., Stewart, H. and Ragge, N. K. (2010). Bone morphogenetic protein 7 (BMP7) mutations are associated with variable ocular, brain, ear, palate, and skeletal anomalies. *Hum. Mutat.* 31, 781-787. 10.1002/humu.21280 20506283

[DEV200399C150] Williamson, K. A. and Fitzpatrick, D. R. (2014). The genetic architecture of microphthalmia, anophthalmia and coloboma. *Eur. J. Med. Genet.* 57, 369-380. 10.1016/j.ejmg.2014.05.002 24859618

[DEV200399C151] Williamson, K. A., Rainger, J., Floyd, J. B., Ansari, M., Meynert, A., Aldridge, K. V., Rainger, J. K., Anderson, C. A., Moore, A. T., Hurles, M. E. et al. (2014). Heterozygous loss-of-function mutations in YAP1 cause both isolated and syndromic optic fissure closure defects. *Am. J. Hum. Genet.* 94, 295-302. 10.1016/j.ajhg.2014.01.001 24462371 PMC3928658

[DEV200399C153] Yamada, R., Oguri, A., Fujiki, K., Shirahige, K., Takezoe, H., Takahashi, N. and Kanai, Y. (2021). Single-cell transcriptional analysis reveals developmental stage-dependent changes in retinal progenitors in the murine early optic vesicle. *Biochem. Biophys. Res. Commun.* 543, 80-86. 10.1016/j.bbrc.2021.01.043 33548738

[DEV200399C154] Yamamoto, Y., Stock, D. W. and Jeffery, W. R. (2004). Hedgehog signalling controls eye degeneration in blind cavefish. *Nature* 431, 844-847. 10.1038/nature02864 15483612

[DEV200399C155] Zenteno, J. C., Buentello-Volante, B., Quiroz-González, M. A. and Quiroz-Reyes, M. A. (2009). Compound heterozygosity for a novel and a recurrent MFRP gene mutation in a family with the nanophthalmos-retinitis pigmentosa complex. *Mol. Vis.* 15, 1794-1798. 19753314 PMC2742641

[DEV200399C159] Zhang, W., Mulieri, P. J., Gaio, U., Bae, G.-U., Krauss, R. S. and Kang, J.-S. (2009). Ocular abnormalities in mice lacking the immunoglobulin superfamily member Cdo. *FEBS J.* 276, 5998-6010. 10.1111/j.1742-4658.2009.07310.x 19754878

[DEV200399C156] Zilova, L., Weinhardt, V., Tavhelidse, T., Schlagheck, C., Thumberger, T. and Wittbrodt, J. (2021). Fish primary embryonic pluripotent cells assemble into retinal tissue mirroring in vivo early eye development. *eLife* 10, e66998. 10.7554/eLife.66998 34252023 PMC8275126

